# An exploratory study of food addiction in Indian youth

**DOI:** 10.1186/s40337-021-00386-9

**Published:** 2021-03-06

**Authors:** Tamoghna Ghosh, Siddharth Sarkar, Aman Tilak, Kanwal Preet Kochhar

**Affiliations:** 1 All India Institute of Medical Sciences, New Delhi, India; 2 Department of Psychiatry and National Drug Dependence Treatment Centre, AIIMS, New Delhi, India; 3 Department of Physiology, Cognitive Neuro Physiology and Nutrition Laboratory, AIIMS, New Delhi, India

**Keywords:** Nutrition, Obesity, Food addiction, YFAS, Food preference, Indian youth

## Abstract

**Background and aims:**

As the understanding of food addiction increases, there is a need to explore the occurrence of this condition in different population groups. This exploratory study aimed to assess the occurrence of food addiction in a sample of respondents from India using a Hindi version of the Yale Food Addiction Scale (YFAS).

**Methods:**

The Hindi language version of the scale was developed using the back-translation methodology. Subsequently, an online questionnaire-based study was conducted using convenience sampling which presented the Hindi version of YFAS.

**Results:**

From 376 respondents (median age 19 years, 42.8% males), the rate of occurrence of food addiction was 13.3%. Persistent desire or repeated unsuccessful attempts to quit was the most common symptom domain endorsed. The weight (median 67 kg versus 60 kg) and BMI (median 25.89 kg/ m^2^versus 23.04 kg/ m^2^) were higher in the food addiction group as compared to the non-food addiction group.

**Conclusions:**

Despite the limitations of potential selection bias, this exploratory study suggests that food addiction may be present in a proportion of young aged Indians. The association of food addiction with higher weight and BMI suggests propensity to develop metabolic syndrome, and the need to evaluate interventions that could modify phenomenological expression of food addiction.

**Supplementary Information:**

The online version contains supplementary material available at 10.1186/s40337-021-00386-9.

## Plain English summary

This exploratory study aimed to assess the occurrence of food addiction in a sample of respondents from India using a Hindi version of the Yale Food Addiction Scale (YFAS). The Hindi language version of the scale was developed using the back-translation methodology and subsequently the questionnaire-based study was conducted online. From 376 respondents the rate of occurrence of food addiction was 13.3%. Persistent desire or repeated unsuccessful attempts to quit was the most common symptom domain endorsed. The weight and BMI were higher in the food addiction group as compared to the non-food addiction group. This suggests that food addiction may be present in a proportion of young aged Indians and proper timely intervention can reduce food addiction.

## Introduction and background

In the recent decades, food addiction as a construct has gathered scientific attention [1, 2]. Addiction to food stuff which is rich in fats, sugars and salts have been conceptualized as a condition akin to addiction to other hedonic psychoactive substances like alcohol and tobacco. The conceptualization of food addiction has been proposed given the scenario of some individuals having cravings towards some food stuff, and excess consumption occurs despite having adverse consequences like obesity. Phenomenologically, some of the criteria for addiction to substances have been found with regards to hyper-palatable varieties of food [3], moving forward the thought that food addiction as a construct has equivalence with substance use disorders. Neurobiological research seems to highlight the commonalities in neural pathways involved in food addiction and substance use disorders [4, 5]. Food addiction has specific connotations with respect to obesity which often results from the imbalance of food intake and energy expenditure [1, 6].

In countries like India, with relative abundance of food in recent years, and reduction in physical exercise, the rates of obesity have been on the rise. Obesity itself has been associated with the occurrence of metabolic syndrome which becomes a risk factor for diabetes mellitus and dyslipidemia. The outcomes of obesity and management of concomitant dyslipidemia and diabetes mellitus becomes challenging with the presence of uncontrolled craving for food and excessive consumption behaviors [7–9]. Hence food addiction as a construct seems to have implications for outcomes of non-communicable diseases [10], which themselves has become grave challenges in the Indian healthcare system. The construct of food addiction has been debated, as to whether it has been a tenable and valid diagnosis, and what would be the psychological, biological and behavioral underpinnings of such a diagnosis [11, 12]. However, this construct has been an active area of research and considerable evidence has been accumulated on the expression, genesis and correlates of the condition [13, 14].

Food addiction, like any other psychiatric condition, needs some structured and systematic manner of assessment. Various aspects of food addiction can be captured through the application of questionnaire-based scales and instruments. Among the questionnaire-based instruments assessing food addiction, the Yale Food Addiction Scale (YFAS) is probably the most commonly used one. A meta-analysis has been done to assess the prevalence of food addiction in the population, which has synthesized results from 25 studies with 196,211 participants [15]. The prevalence rates of food addiction have been reported to range from 5.4% to more than 50% across different studies which have used the YFAS. The prevalence rates, from this systematic review involving close to 200,000 subjects, suggests that food addiction is higher in women and those from the clinical population, rather than the general population. There is only one study from India which reported the rate of food addiction to be 32.5%, using the English version of YFAS [16]. A wider application of YFAS would need suitable translation into different languages. Hindi, is probably the third most commonly spoken language worldwide  [17], and hence translation of YFAS questionnaire into Hindi is likely to facilitate assessment of a number of individuals. We aimed to conduct and exploratory study to translate the YFAS into Hindi language and to find out the prevalence of food addiction in an Indian population. We also aimed to assess the relationship of food addiction and BMI.

## Methodology

### Questionnaire and translation

Hindi translation of YFAS was developed through the back-translation methodology. The YFAS comprises 25 single choice items. The questionnaire looks at past year eating habits and focuses on sweets, starches, salty snacks, fatty foods, sugary drinks or any other food stuff which the individual considers as problematic. The questionnaire has 16 Likert rated items, 9 yes/no rated items, one multiple choice item, and one open ended question. Based upon the responses, the individual is classified into having either food addiction or not. The instrument has been used in general populations, and clinical populations (like those with diabetes mellitus or obesity).

The initial phase of this exploratory study involved translation of the YFAS into Hindi language by two bilingual doctoral level experts who had previously not seen the instrument. The translation was aimed at semantic translation rather than literal translation. Consensus was achieved from the experts on the words that differed in the instrument to have a consistent instrument. The example food items were modified to include the common food products in the region. The Hindi version was then translated into English by two other bilingual experts, and the consensus version was sent to the original developer of the scale to ascertain conceptual validity for the items and the scale.

### Online survey

The application of the Hindi version in a larger sample was done through an online survey, cognizant of the ongoing pandemic. A GoogleForms questionnaire was developed. Age, gender, educational status, employment, current weight and height were enquired into. Body mass index (BMI) was computed based upon the reported height and weight. The questionnaire presented the Hindi items of the YFAS. Adult participants who were willing to provide online consent were recruited to participate in the study. Participants were recruited by sending emails and through WhatsApp to the contacts of the investigators. Participants were free to send the questionnaire further to their contacts. Data collection was done for a period of 5 days in the month of October 2020. A formal sample size estimation was not done, but it was aimed to have more than 200 responses.

The responses from the participants were exported into excel files, and then were imported into SPSS version 22.0 (IBM Corp, Armonk, NY). Scoring of the YFAS was done as per the procedure used by Gearhardt et al.  [18], which evaluates presence of substance dependence criterion. The diagnosis of food addiction is based upon presence of at least three of seven criteria, along with presence of impairment criterion. Descriptive statistics were used to represent the nominal, ordinal and scale data. Normality was assessed using Kolmogorov Smirnov test. Internal consistency of the test was measured using Cronbach alpha. The relationship of the presence of a diagnosis of food addiction with gender, age, weight, height and BMI was assessed using suitable non-parametric tests (chi square and Mann Whitney U test). A *p* value of less than 0.05 was considered and missing value imputation was not done.

## Results

We received a total of 376 usable consenting responses (total responses were 396, 5 responded not consenting to participate, 15 reported outside of possible range weight or height values). Among them, 161 (42.8%) were males, 214 were females (56.9%) and 1 identified as ‘others’ (0.3%). A total of 364 respondents were students (96.8%). The median age of the sample was 19 years (interquartile range of 18 to 20 years). The median (interquartile range [IQR]) weight, height and BMI of the sample was 60 kg (50 kg, 70.5 kg), 162 cm (155 cm, 173 cm) and 23.2 kg/ m^2^ (19.8 kg/ m^2^, 27.1 kg/ m^2^) respectively.

The responses to individual questions of the Hindi version of YFAS are presented in Table 1. Among the Likert rated questions, eating more than planned was most frequently endorsed. Among the dichotomous, yes-no type rated questions, wanting and trying to cut down certain types of foods were most commonly endorsed. The Cronbach alpha for the scale was 0.897.

**Table 1:** Responses to the YFAS questionnaire items

S No	Item	Response Mean (SD) or n (percentage)
1	Eating much more than planned	2.73 (1.28)
2	Continuing to consume certain foods even though no longer hungry	2.36 (1.48)
3	Eat to the point where I feel physically ill	1.28 (1.87)
4	Worry about Not eating certain types of food or cutting down on certain types of food	1.89 (1.61)
5	Spend a lot of time feeling sluggish or fatigued from overeating	2.16 (1.52)
6	Find self constantly eating certain foods throughout the day	1.73 (1.70)
7	Going out of the way to obtain certain food when they are not available	2.07 (1.48)
8	Missing out on other activities	1.43 (1.84)
9	Dealing with negative feelings of overeating	1.49 (1.79)
10	Avoided professional/ social situations due to fear of overeating	1.23 (1.90)
11	Avoided professional/ social situations as could not consume certain food there	1.42 (1.80)
12	Withdrawal symptoms on cutting down or stopping certain foods	1.38 (1.85)
13	Consumed food to prevent feelings of anxiety, agitation or other physical symptoms	1.55 (1.76)
14	Elevated desires or urges to consume certain types of foods	1.99 (1.49)
15	Significant distress	1.73 (1.73)
16	Significant problems in ability to function effectively	1.74 (1.70)
17	Food consumption has caused significant psychological problems	61 (16.22%)
18	Food consumption has caused significant physical problems or made a physical problem worse	129 (34.31%)
19	Kept consuming though having emotional and/or physical problems	95 (25.27%)
20	Over time, need to eat more and more to get the desired feeling	103 (27.39%)
21	Same amount of food does not reduce negative emotions or increase pleasurable feelings as it used to	104 (27.66%)
22	Want to cut down or stop eating certain kinds of food.	249 (66.22%)
23	Tried to cut down or stop eating certain kinds of food	255 (67.82%)
24	Been successful at cutting down or not eating these kinds of food	194 (51.6%)
25	Number of times in past year tried to cut down or stop certain foods	2.88 (1.69)

Responses are in the form of Never (1)/ Once a month (2)/ 2–4 times a month (3)/ 2–3 times a week (4)/ 4 or more times or daily (5) (question 1 to 16) or Yes / No (question 17 to 24), or 1 or fewer times (1)/ 2 times (2)/3 times (3)/ 4 times (4)/ 5 or more times (5) (question 25)

The domains endorsed by the participants are presented in Table 2. The most common domains of food addiction that were endorsed by the participants were persistent desire or repeated unsuccessful attempts to quit, and tolerance. It was seen that 13.3% of the sample had a diagnosis of food addiction.

**Table 2:** Domains of YFAS (*n* = 376)

Domain	Number of participants satisfying the criteria (percentage)
Taken in larger amount and for longer period than intended	56 (14.9%)
Persistent desire or repeated unsuccessful attempts to quit	334 (88.8%)
Much time/activity to obtain, use, recover	97 (25.8%)
Important social, occupational, or recreational activities given up or reduced	76 (20.2%)
Use continues despite knowledge of adverse consequences	95 (25.3%)
Tolerance	162 (43.1%)
Withdrawal symptoms	49 (13.0%)
Clinically significant impairment or distress	58 (16.4%)
Diagnosis of food addiction	50 (13.3%)

Those with a diagnosis of food addiction did not differ from with those without a diagnosis of food addiction on gender, age and height. However, food addiction was associated with weight and BMI. The weight (median 67 kg versus 60 kg, Mann Whitney U = 5991, *p* = 0.013) and BMI (median 25.89 kg/ m^2^ versus 23.04 kg/ m^2^, Mann Whitney U = 5439.5, *p* = 0.011) were higher in the food addiction group.

## Discussion

The present exploratory study suggests that the food addiction in this predominantly young adult population was present in around 13.3% of the participants. Also, presence of food addiction was seemingly more common in those with higher weight and BMI. The rate of occurrence of food addiction was lesser than the weighted mean prevalence of 19.9% reported by the meta-analysis by Pursey et al. [15]. The occurrence of food addiction was also lower than the other study from Indian population which reported a rate of 32.5% [16]. The occurrence rate is similar to studies from other parts of the world, for example, Eichen et al., [19] reported a rate of 15.2% while Gearhardt et al. [18] reported rates of 11.4%. Even lower prevalence rates have also been reported in some other studies, for example rates of 8.2 and 7.8% by Mason et al., [20] and Meule et al. [21] respectively.

The present study did not find a relationship between age or gender and food addiction. This is at a variance from previous other studies which have suggested that greater age and female gender was associated with food addiction [15]. There is some literature, including nationally representative samples, which suggest that gender differences may not apply for food addiction [22–24]. It is possible that the culturally determined expectations and eating attitudes may influence how options are perceived, appraised and responded to. There is some evidence to suggest that women from India and other Asian countries have less drive for thinness than Western countries [25], however there is evidence to the contrary as well [26]. The study failed to replicate higher rates of food addiction in the younger population, as the sample largely consisted of younger individuals (the interquartile range of age was 18 to 20 years).

The association of food addiction and weight and BMI as suggested in the present sample are in similar lines with the other studies [15, 27]. The causality of the association has been explored, and it has been suggested that while food addiction may lead to uncontrolled eating and consequently greater weight gain. Food addiction also has been implicated in lack of efficacy of weight loss interventions [27]. The median age of those who had food addiction in this study fell in the overweight range, suggesting an at-risk group, where gradual increase of BMI was likely to lead to obesity and consequent medical conditions. The overall rates of food addiction seems to be higher in the group of obese individuals [15].

Among the symptoms of food addiction, the most common symptoms endorsed pertained to persistent desire or repeated unsuccessful attempts to quit. Other nationally representative studies have also suggested this item to be most common [23, 24]. However, certain other items like giving up important social, occupational, or recreational activities were endorsed quite less frequently. The implication of this phenomenology is that individuals might not have dysfunction in their different functional domains, though they might find themselves unable to quit specific food stuff. This probably highlights that the adverse consequences of food addiction may not be immediate, but in the long-term as individuals are unable to control their food intake.

The findings of the present study have some implications. The occurrence of this condition in the population as ascertained using a commonly used questionnaire, suggests that food addiction can be delimited, furthering research on the construct. The association with high BMI may help direct query towards food consumption habits during the routine clinical encounter. Clinically, many patients may present with binge eating, though cognizance has to be made that though there can be overlap in presentation, not all individuals who have binge eating would have food addiction [28]. From a policy perspective, attention towards foodstuffs that are consumed to satisfy craving beyond the point of nutritional relevance may help to make informed choices by the consumers. This may look premature in Indian context given the extent of under-nutrition in the country, but childhood and adult obesity is a rising concern and preventive measures may help to address the genesis of several non-communicable diseases.

Some limitations of the present study merit attention. The sampling was through contacts through email and social media of the contacts of investigators, resulting in possible selection biases. Also, the sample consisted mainly of the younger population, and may not represent the findings from other age groups. A more representative sample from different strata, socio-economic groups, digital literacy levels and geographical regions would have given more generalizable findings. Additionally, the study did not manually check for weight and height and relied on the self-reported values, which could just have been fair guess. Additional measurements like body impedance were not conducted in the study. We did not assess the convergent validity, divergent validity, concurrent validity or test-retest reliability of the Hindi version of YFAS questionnaire. We did not look at temporal stability of food addiction, and did not look at other related determinants like physical activity, body fat composition and calorie or fat intake. The findings of the study may be considered as preliminary due to the exploratory nature of the study, and more rigorous research is required to draw firm conclusions, especially prevalence of food addiction in the population.

## Conclusion

To conclude, the present exploratory study suggests that food addiction may be present in a considerable minority of Indian young adult population, though the potential selection biases may limit generalizability. Food addiction is associated with higher weight and BMI. Future studies may look at the relationship of food addiction and trajectory of development of obesity. The occurrence of food addiction in different populations can be ascertained. Research in the neurobiological understanding of appraisal of specific food products, and methods to control craving would be helpful to devise better treatment strategies.

## Supplementary Information

**Additional file 1.**

## Data Availability

The data would be made available by the authors on request.

## Abbreviations

BMI: Body Mass Index
YFAS: Yale Food Addiction Scale
