# Supplementary material for: An exploratory study of food addiction in Indian youth
Source: J Eat Disord. 2021 Mar 6;9:32. doi: 10.1186/s40337-021-00386-9 (PMC7937298; doi:10.1186/s40337-021-00386-9)
Supplement: Supplementary file 1 — Additional file 1. [file 40337_2021_386_MOESM1_ESM.pdf]

Yale Food Addiction Scale (Hindi version)

येल भोजन की लत का पैमाना (हिंदी रूपांतरण)

यह प्रश्नावली पिछले वर्ष में खाने की आदतों के बारे में है। कुछ लोगों को कभी-कभी अपने खाद्य पदार्थों के सेवन को नियंत्रित करने में कठिनाई होती है, जैसे:

- मीठा खाना जैसे आइसक्रीम, बिस्किट, चॉकलेट
- चावल या मैदे और आटे से बनी चीजें जैसे ब्रेड
- नमकीन खाद्य पदार्थ जैसे चिप्स, नमकीन
- वसायुक्त भोजन जैसे मांस, पिज्जा
- मीठा पेय जैसे सोडा

जब खाने की चीजों के बारे में निम्नलिखित प्रश्न पूछे जाते हैं, तो कृपया उन खाद्य पदार्थों के बारे में सोचें जो खाद्य समूह या किसी अन्य खाद्य पदार्थ में सूचीबद्ध हैं जिससे पिछले एक साल में समस्या आई है|

	पिछले 12 महीनों में	कभी नहीं	महीने में एक बार	महीने में 2-4 बार	सप्ताह में 2-3 बार	सप्ताह में 4 या अधिक बार या हर रोज
1.	मैंने पाया है कि जब मैं कुछ खाने की चीजों को खाना शुरू करता/ करती हूँ तो जरूरत से ज्यादा खा लेता/ लेती हूँ।	0	1	2	3	4
2.	मैंने पाया है कि मैं कुछ खाने की चीजों को भूख पूरी होने के बाद भी खाता रहता/ खाती रहती हूँ।	0	1	2	3	4
3.	मैं उस हद तक खाता रहता/ खाती रहती हूँ जब तक मुझे तबियत खराब होने का एहसास नहीं होता।	0	1	2	3	4
4.	कुछ तरह की चीज़ें न खाने से या उनमें कटौती करने से मैं परेशान हो जाता/ जाती हूँ।	0	1	2	3	4

Yale Food Addiction Scale (Hindi version)

5.	मेरा अधिकतर समय जरूरत से ज्यादा खाने के बाद हुई सुस्ती या थकान में बीत जाता है।	0	1	2	3	4
6.	मैं अपने आप को पूरे दिन लगातार एक ही प्रकार का खाना खाते हुए पाता/ पाती हूँ ।	0	1	2	3	4
7.	कुछ खाने की चीज़ें उपलब्ध न होने पर, मैं प्राप्त करने के लिए उन्हें बाहर से लेकर आता/ आती हूँ जैसे कि बाजार/दुकान से उनको खरीदने जाऊँगा/ जाऊँगी जबकि मेरे पास घर पर दूसरे विकल्प होंगे।	0	1	2	3	4
8.	ऐसा समय भी आया है जब मैंने खाने की चीजों को इतनी बार खाया है या इतना ज्यादा खा लिया है कि मैंने काम करने, परिवार के साथ समय बिताने या अपने रूचिकर कार्य करने की बजाय इन चीजों को खाना शुरू कर दिया।	0	1	2	3	4
9.	ऐसा भी कई बार हुआ है कि मैंने अपना समय, अपने काम, दोस्तों, परिवार या रूचिकर कार्यों में न लगाकर ज्यादा खाने से उत्पन्न हुई नकारात्मक भावनाओं से निपटने में बिता दिया है।	0	1	2	3	4
10.	ऐसा कई बार हुआ है जब मैं नौकरी संबंधी एवं सामाजिक मौकों पर नहीं गया/ गयी हूँ क्योंकि वहां मैं बहुत ज्यादा खा लेता/ लेती।	0	1	2	3	4
11.	ऐसा अवसर आया है जब मैं औपचारिक अथवा/तथा सामाजिक अवसरों पर नहीं गया/ गयी क्योंकि मैं वहां खाना नहीं खा पाता/ पाती।	0	1	2	3	4
12.	जब मैंने कुछ खाने की चीजों का सेवन बंद किया तो मुझे बेचैनी, घबराहट या उत्तेजना जैसे शारीरिक लक्षण हुए (कृपया इनमें चाय, कॉफी, सोडा या ऊर्जा देने वाले पेय शामिल ना करें)।	0	1	2	3	4

13.	मैंने बेचैनी, उत्तेजना और घबराहट जैसे शारीरिक लक्षण होने से रोकने के लिए खाने की चीजों का सेवन किया है।	0	1	2	3	4
14.	मैंने पाया है कि कुछ खाने की चीजों में कटौती करने या उन्हें बंद करने से उन्हें खाने की तलब (इच्छा) और बढ़ जाती है।	0	1	2	3	4
15.	खाने की चीजों के प्रति मेरे व्यवहार से मेरे जीवन में काफी परेशानियां है।	0	1	2	3	4
16.	खाने की चीजों और उन्हें खाने की वजह से मुझे अपनी कार्य क्षमता (दिनचर्या), नौकरी, सामाजिक, पारिवारिक गतिविधियों, या स्वास्थ्य संबंधित समस्याओं का सामना करना पड़ता है।	0	1	2	3	4

	पिछले 12 महीनों में	नहीं	हाँ
17.	कुछ खाने की चीजों के सेवन से मुझे उदासी, चिंता, घबराहट, आत्मदोष जैसी गंभीर मानसिक परेशानियां हुई है।	0	1
18.	कुछ खानें की चीजों के सेवन के कारण मुझे शारीरिक परेशानियां उत्पन्न हुई है या वे और बदतर हुई है।	0	1
19.	शारीरिक तथा/अथवा मानसिक परेशानियां होने के बाद भी मैंने उसी मात्रा में या उससे अधिक मात्रा में खाना खाया है।	0	1
20.	मैंने पाया है कि समय बीतने के साथ-साथ मुझे अपनी इच्छानुसार नकारात्मक भावनाओं में कमी या सकारात्मक भावनाओं में बढ़ोत्तरी करने के लिए इन चीजों की अधिक जरूरत पड़ती है।	0	1
21.	मैंने पाया है कि उतनी ही मात्रा में खाने से न तो मेरी नकारात्मक भावनाएं कम होती है और न ही पहले जितनी सुखद भावनाओं की अनुभूति होती है।	0	1
22.	मैं कुछ प्रकार के खाने की चीजों को खाना बंद या उन्हें कम खाना चाहता / चाहती हूँ।	0	1

23.	मैंने कुछ प्रकार के खाने के चीजों को कम करने की कोशिश की है।	0	1
24.	मैं इन खाने के चीजों को कम करने या बंद करने में सफल हुआ / हुई हूँ।	0	1

25.	पिछले एक साल में आपने कितनी बार इन खाने की चीजों को छोड़ने की कोशिश की है?	1 बार या कम	2 बार	3 बार	4 बार	5 बार या अधिक

26.	कृपया निम्नांकित उन खानों के नाम पर गोला लगाएं, जिनसे आपको समस्याएं हैं

आइसक्रीम	चाकलेट	सेब	मिठाई	पालक
बिस्कुट	केक	टॉफी	डबलरोटी (ब्रेड)	समोसा
सलाद	पास्ता	अंगूर	चावल	मठरी
चिप्स	जलेबी	तले आलू (फ्रेंचफ्राइज)	गाजर	चिकन
केला	मांस	बर्गर	कनान	पिज्जा
कोल्ड ड्रिंक	नमकीन	पकौड़ा	पोहा	कुछ और

27. कृपया कोई और खानों की चीजों की सूची दें जो ऊपर अंकित नहीं की गई है:
